# Bond–Slip Relationship between Sand-Coated Polypropylene Coarse Aggregate Concrete and Plain Rebar

**DOI:** 10.3390/ma15072643

**Published:** 2022-04-03

**Authors:** Heru Purnomo, Mochamad Chalid, Gandjar Pamudji, Taufiq Wildan Arrifian

**Affiliations:** 1 Department of Civil Engineering, Universitas Indonesia, Depok 16424, Indonesia; wildantrf@gmail.com; 2 Department of Metallurgical and Materials, Universitas Indonesia, Depok 16424, Indonesia; chalid@eng.ui.ac.id; 3 Department of Civil Engineering, Universitas Jenderal Soedirman, Tengah 53122, Indonesia; gandjar.pamudji@unsoed.ac.id

**Keywords:** polypropylene coarse aggregate, plain rebar, bond strength, bond–slip relationship

## Abstract

Recycled plastic waste as an aggregate in concrete mixtures is one of the important issues in the construction industry since it allows the reduction of building weight and has beneficial effects on the environment. In addition, the bonding ability of this kind of lightweight concrete to reinforcement is also a prerequisite as a composite material in forming reinforced concrete structures. Therefore, in this study, the bond of plain rebar embedded in artificial lightweight aggregate concrete made from polypropylene plastic waste coated with sand was investigated. A pull-out test of nine group specimens was conducted to study the bond strength of 10 mm, 12 mm, and 16 mm diameter plain rebar embedded in polypropylene plastic waste coarse aggregates lightweight concrete (PWCAC), failure mode, and bond stress–slip relationship. The test results show that the bond–slip relationship and bond strength depend mainly on the bar diameter for PWCAC. Meanwhile, for all PWCAC specimens tested, the pull-out failure modes were observed. A bond equation for PWCAC was formulated by performing a regression analysis on the experimental results and afterward was combined with an existing bond–slip equation for normal concrete to have the bond–slip formulation for the lightweight concrete studied. The comparison between the model and experimental results indicates a close agreement.

## 1. Introduction

The bond between the rebar and the surrounding concrete is one of the most important aspects of a reinforced concrete, where the effectiveness depends on the force transfer mechanism at the interface zone. The bond mechanism of plain rebar differs from that of deformed rebar embedded in concrete. In plain rebar, the transfer of force by adhesion is caused by the chemical bonding between the reinforcement and the concrete before bar slip and the friction forces that occur after the rebar slips due to the loss of chemical bonds at a low level of stress. In comparison, deformed bars must rely on the transfer of a large proportion of these forces by mechanical interlocking between the rib and the surrounding concrete [1,2]. 

Generally, a bond stress–slip relationship can reflect bond behavior between reinforcement and concrete. The four test methods used to describe the bond strength–slip relationship and to evaluate bond properties between steel and concrete specified in ACI 408R-03 [3] are called pull-out, beam end, beam anchorage, and beam splice. Pull-out testing is the most widely used method because of the ease of fabrication and the testing procedure.

The bond–slip behavior of plain rebar has been studied since the introduction of reinforced concrete. Various factors affect the bond performance of plain bars, including the influence of bonded length, bar shape and size, concrete strength and pull-out loading rate [3,4], bond stress distribution along the length of the bar in concrete [5], lateral loading [6], and bonding characteristics of the plain bar in low-strength concrete [7], which have also been studied by pull-out test. Feldman and Bartlett [8] assessed the variability of the bond strength of plain bars embedded in normal concrete and derived a bond stress–slip relationship, whereby the bond stress and slip at any point along the embedded reinforcement can be obtained for certain boundary conditions [5]. In addition, experiments were carried out on the bond behavior between plain bars and special concrete such as high-performance concrete [9], recycled aggregate concrete [10,11], and lightweight aggregate concrete [12,13,14].

In recent trends, research activities that integrate concrete technology and the environment have become a worldwide concern. For example, the use of plastic waste as an aggregate in producing lightweight concrete has been conducted by several researchers including Purnomo et al. [15], Pamudji et al. [16], and Abu-Saleem et al. [17], related to the plastic waste of polypropylene (PP); Al Bakri et al. [18], Abeysinghe et al. [19], and Abu-Saleem et al. [17], related to high-density polyethylene; Choi et al. [20], Frigione [21], Islam et al. [22], Abu-Saleem et al. [17], and Alqahtani [23], related to polyethylene terephthalate; and Lakshmi and Nagan [24], Arora [25], Ali et al. [26], Ahmad et al. [27], and Ullah et al. [28] in relation to electronic wastes.

British Design Code BS 8110 is widely used as current national codes of practice for reinforced concrete structures in several countries in Africa, such as Nigeria and Uganda [29]. Two reinforcing steel bars are classified in the BS 8110. One of them is plain round bars of a characteristic yield strength of 250 N/mm^2^. Based on this fact, the plain rebar was used in this study.

The lack of information regarding the bond characteristics between plain rebar and lightweight concrete made from PP waste coarse aggregates (PWCA) is one of the main obstacles to its acceptance in the construction industry. Thus, the bond–slip behavior of plain rebar in this plastic aggregate lightweight concrete is not fully understood, and further research is still needed. Although Pamudji et al. [30] have investigated the bond–slip behavior of a steel bar embedded in PWCA concrete (PWCAC), no bond strength formulation or bond strength–slip relation was proposed in the study. Therefore, this study is a continuation of their research and emphasizes experimental studies to evaluate the bond strength–slip relation of plain rebar embedded in lightweight concrete using coarse aggregates made from sand-coated polypropylene plastic waste. The evaluation was measured by performing a pull-out test, RILEM TC-RC 6 standard [31]. The pull-out test was conducted to determine the bond strength between plain rebar and PWCAC where the influence of the compressive strength of the concrete and the size of the diameter of the reinforcement were studied. The next step was to analyze the ultimate bond strength which will be introduced to the bond–slip relationship. A bond strength equation for PWCAC was formulated and then combined with an existing bond–slip equation to have the bond–slip formulation for the lightweight concrete studied

## 2. Materials and Methods

### 2.1. Materials

#### 2.1.1. Cement and Superplasticizer

The binder material used in this study is Portland Composite Cement (PCC) produced by PT. (Indocement Tunggal Prakarsa, Bogor, Indonesia), where production standards of SNI 15-7064-2014, ASTM C595-13, and EN 197-1:2011 are satisfied. From the supplier information, the PCC has a specific gravity range of 3.00–3.05. The superplasticizer (SP) was applied as the admixture material for all w/c ratio to enhance the fresh concrete workability with a 1.18 to 1.2 specific gravity at 27 °C.

#### 2.1.2. Fine Aggregate and PP Waste Coarse Aggregate

The fine aggregate used in this study is river sand (RS) as a constituent of lightweight concrete. The physical properties, such as specific gravity and water absorption, were tested on the material according to the ASTM C 128 standard, the bulk density according to ASTM C 29, and the granular fineness modulus according to ASTM C 33, and are presented in Table 1.

**Figure 1:**
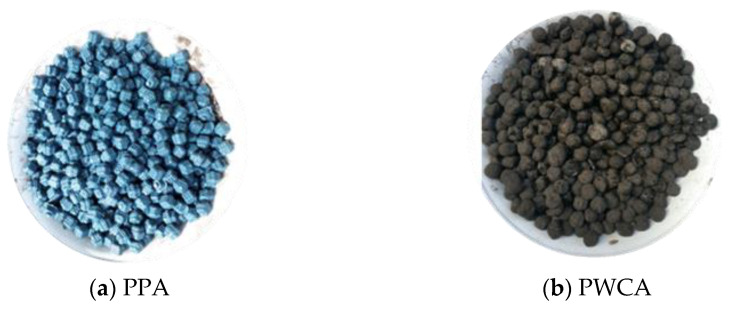
Polypropylene waste coarse aggregate.

The coarse aggregate used as a constituent of lightweight concrete in this study is a coarse aggregate made from PP plastic waste. Plastic waste that has been shredded is fed into an injection machine to be shaped similarly to natural coarse aggregate and is referred to as plain plastic aggregate (PPA); it has a size of 7.5–20 mm, as developed by Pamudji et al. [32], and is shown in Figure 1. The following process is to coat the surface of the PPA with river sand to increase the adhesion with the cement paste, and the thickness of the sand layer is increased by approximately 1 mm [15,16]. Testing the physical properties of PWCA as lightweight aggregates refers to the standard ASTM C330 [33]/SNI 2461 [34] as presented in Table 1.

#### 2.1.3. Reinforcement Steel

**Figure 2:**
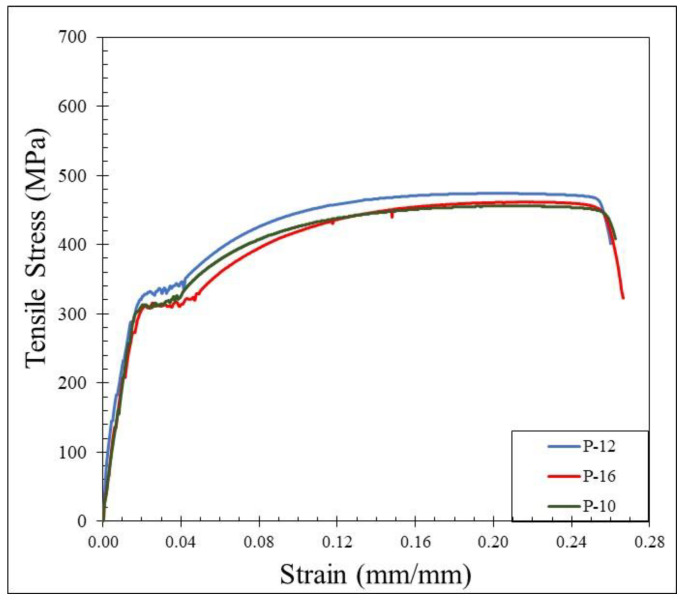
Stress–strain relationship of plain bars.

Plain reinforcing bars with 10 mm, 12 mm, and 16 mm diameters were selected in the bond test. The mechanical properties of the rebar are listed in Table 2 and shown in Figure 2.

### 2.2. Mix Proportions

Three mix proportions of PWCAC were designed to prepare specimens based on water–cement ratio and varied as to PWCA content, corresponding to a target strength class of 20 MPa to 26 MPa. All the mix proportions, including a normal concrete mix, are presented in Table 3. The specimen prepared with normal concrete (NC23) is used as control specimen.

### 2.3. Designation of Specimens

**Figure 3:**
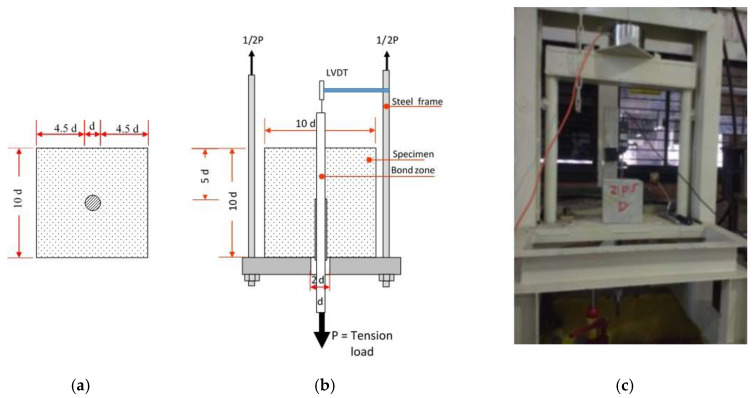
Pull-out test specimens (**a**), setting (**b**), and (**c**) testing equipment.

The experimental investigation with a pull-out test was designed to study the influence of two main parameters, concrete strength and diameter of reinforcement, in the bond strength between a steel bar and lightweight concrete made of sand-coated plastic waste coarse aggregate. A total of 29 pull-out specimens were cast using PWCA with three different concrete mixtures, as presented in Table 3. There were three specimens in each series for testing. Plain rebar with nominal diameters of 10, 12, and 16 mm was then embedded in each PWCA concrete mix with 400 mm of the rebar length protruding out on one end as the free end and 30 mm of the rebar at the other end as the loaded end. This was done to obtain a slip measurement, and so that the rebar could be easily grasped during the pull-out test. The embedded length rebar of all specimens was maintained within five times the reinforcing bar diameter, and the concrete cover was chosen as over 4.5 times the rebar diameter to avoid splitting failure. Polyvinyl chloride (PVC) tubes were used to ensure the un-bonded zone length. The dimensions of the pull-out specimens and setting are shown in Figure 3, where d is the rebar diameter.

### 2.4. Test Method

The dry density and compressive strength of cylindrical concrete with a diameter of 15 cm and a height of 30 cm were tested when the specimen was 28 days old, referring to ASTM C567 [35] and ASTM C39 [36] standards, respectively. In addition, the concrete strength values for all pull-out specimens were tested using the non-destructive method by ultrasonic pulse velocity (UPV). Table 4 and Table 5 present the compressive strength from all specimens tested by the destructive and non-destructive methods.

A hand hydraulic pump was applied as the tension load and the settings for the pull-out test which followed the RILEM TC-RC6 [31] are shown in Figure 3. To determine the bond strength–slip relationship, the load and slip at the free ends of the rebar embedded in the concrete were measured. The hand hydraulic pump monotonically increasing load was applied and read through the load cell display monitor and analog manometer for comparison. One linear variable differential transducer (LVDT) was attached to the rebar. Therefore, the bond strength can be calculated as the normal force divided by the perimeter multiplied by the length of the reinforcement embedded *l_d_* in the concrete, as shown in the following equation.

(1) $$\tau =\frac{P_{u}}{\pi dl_{d}}$$

where *τ* is the bond strength (MPa), *P_u_* is the axial load (N), *d* is the rebar diameter (mm), and *l_d_* is the bond length (mm).

## 3. Results and Discussion

### 3.1. Physical and Mechanical Properties of Concrete

The dry unit weight, destructive, and/or non-destructive values of compressive strength, bond strength, and slip are presented in Table 4, Table 5 and Table 6. From Table 4, it is shown that the dry unit weight value is less than 1840 kg/m^3^, which is included in the classification of lightweight concrete according to SNI-2461 [34] and ASTM C 330 [33]. The test results show that the average compressive strength of concrete at the age of 28 days increased with the decrease in PWCA content in the concrete mixture. The results of the destructive compression test were used to verify those given by the non-destructive UPV test apparatus.

### 3.2. Bond Stress–Slip Behavior

**Figure 4:**
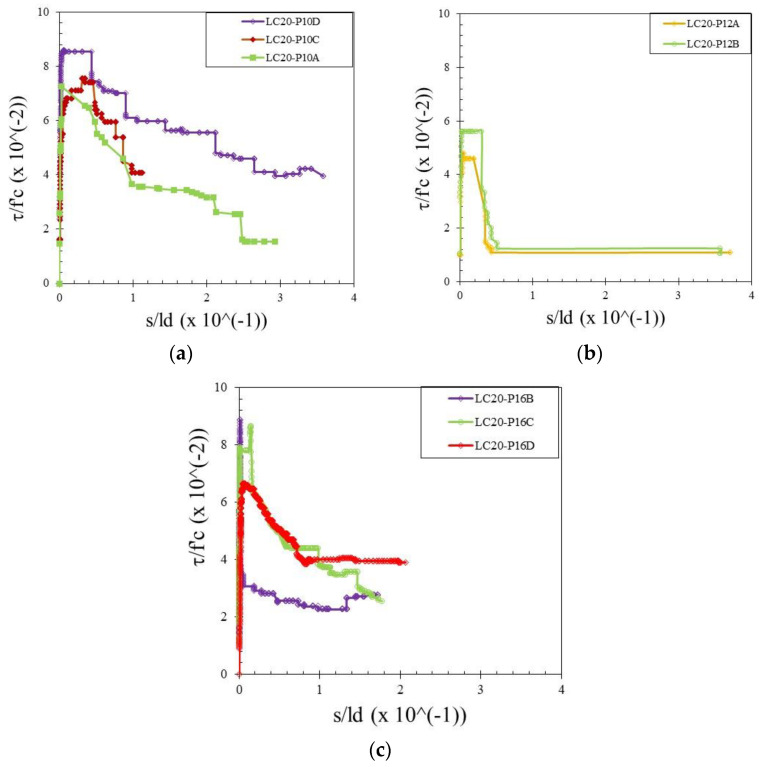
Local bond stress–slip curve for LC20 PWCAC, (**a**) 10 mm plain rebar, (**b**) 12 mm plain rebar, (**c**) 16 mm plain rebar.

**Figure 5:**
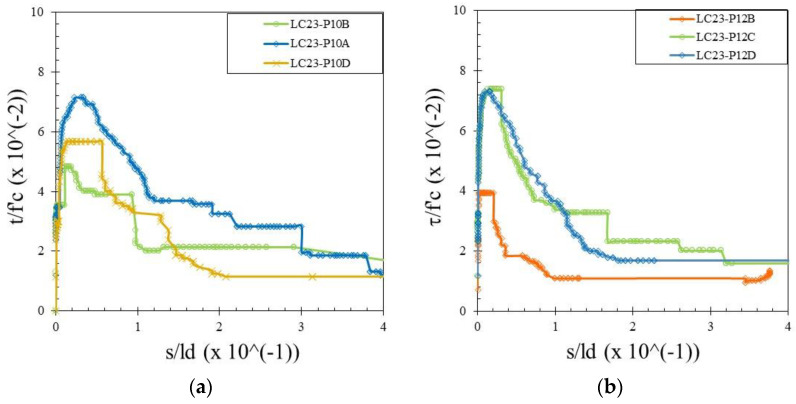

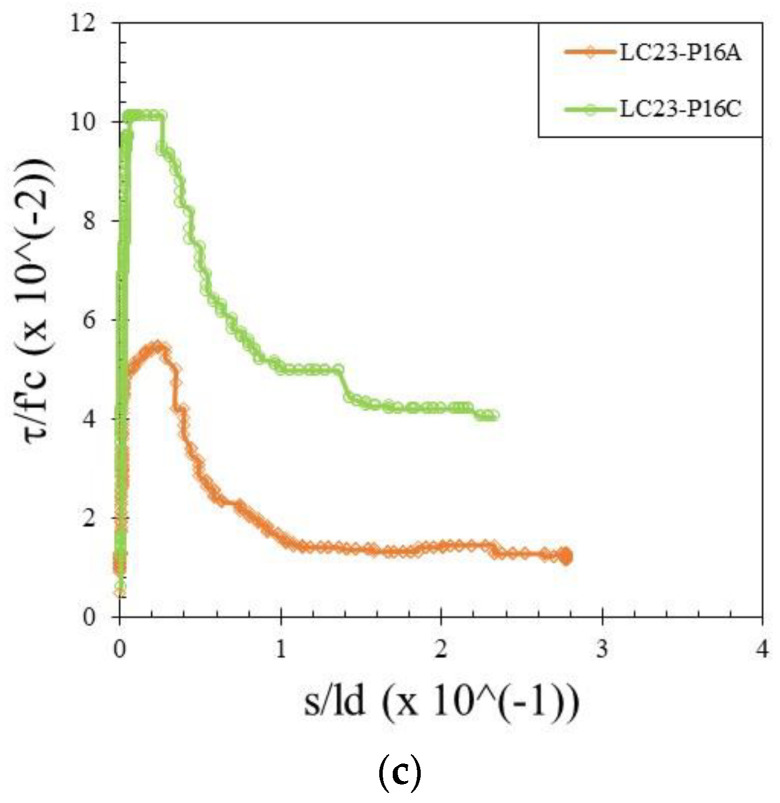
Local bond stress–slip curve for LC23 PWCAC, (**a**) 10 mm plain rebar, (**b**) 12 mm plain rebar, (**c**) 16 mm plain rebar.

**Figure 6:**
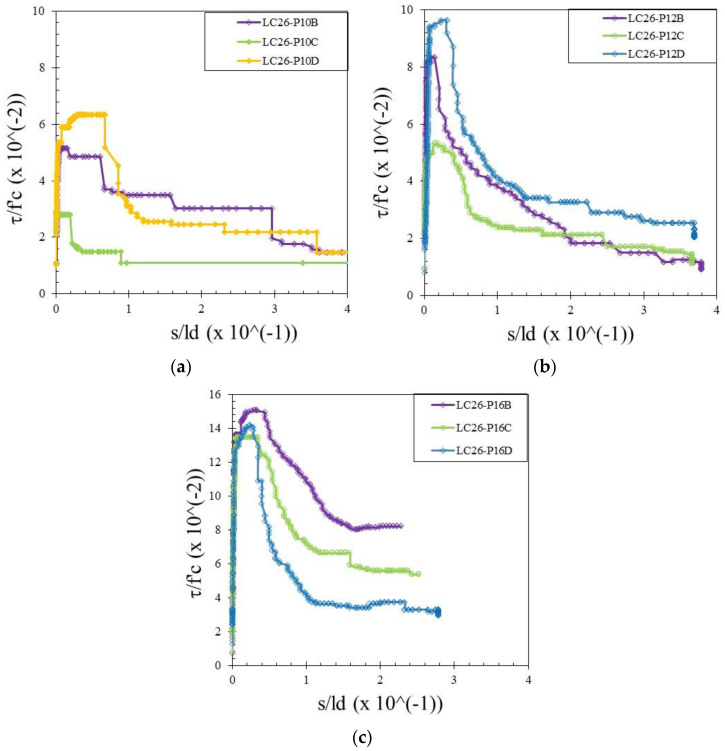
Local bond stress–slip curve for LC26 PWCAC, (**a**) 10 mm plain rebar, (**b**) 12 mm plain rebar, (**c**) 16 mm plain rebar.

**Figure 7:**
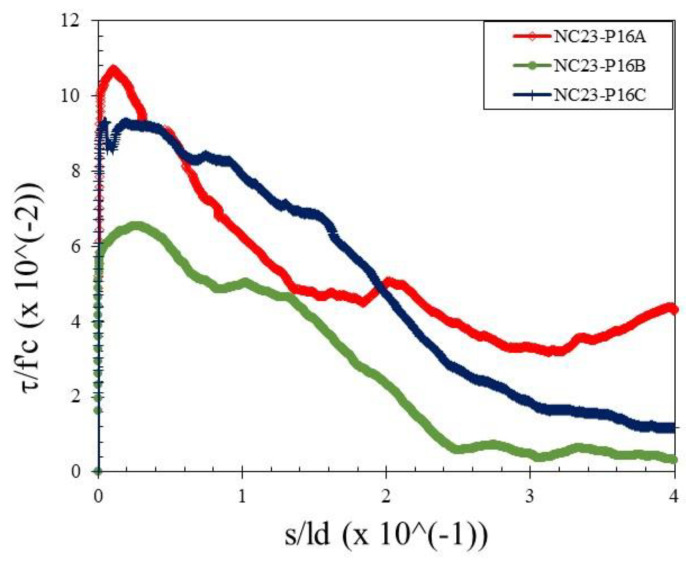
Local bond stress–slip curve for NC23-P16.

The bond stress was calculated according to Equation (1) and the slip was determined by measuring the relative movement of the free end of the rebar to the concrete. Therefore, the bond–slip relationship curve could be drawn directly based on the test data. Bond–slip curves of rebar free ends for specimens with different concrete strengths and rebar diameters are presented in Figure 4, Figure 5 and Figure 6. Figure 7 shows the bond–slip curve from plain rebar embedded in natural aggregate concrete as specified in Table 6. The axes in Figure 4, Figure 5, Figure 6 and Figure 7 were normalized by dividing the bond stress by the concrete compressive strength on the ordinate axis and dividing the slip by the embedded length of rebar on the abscissa axis. The bond–slip curve between plain reinforcement and lightweight concrete from sand coated plastic waste aggregates was similar in shape to that between plain rebar in natural aggregate concrete as obtained by Xing et al. [37] and Cairns [1]. For the same concrete compressive strength group LC23, the bond slip curves for the 16 mm plain bars embedded in PWCAC and NAC were similar in shape, as presented in Figure 5c and Figure 7. A larger measurement capacity of LVDT was used in the NAC test compared to that used in the PWCAC test. In the rising part of the ascending section of the curve, initially the bond strength was very small and the slip was not obvious at the free end of the rebar, while the curve tended to remain linear; Wang et al. [38] termed this condition micro-slip.

Furthermore, the critical stress slip began to occur at the ends of the rebar, which indicates that the adhesion by the concrete to the interface zone began to disappear. The next role was taken over by friction, where the curve started to increase non-linearly until it reached the maximum bond strength, and the slip was still small. However, once the maximum stress was reached, the curve immediately softened with an increasing slip and finally reached a stable residual bond strength.

### 3.3. Failure Mode

**Figure 8:**
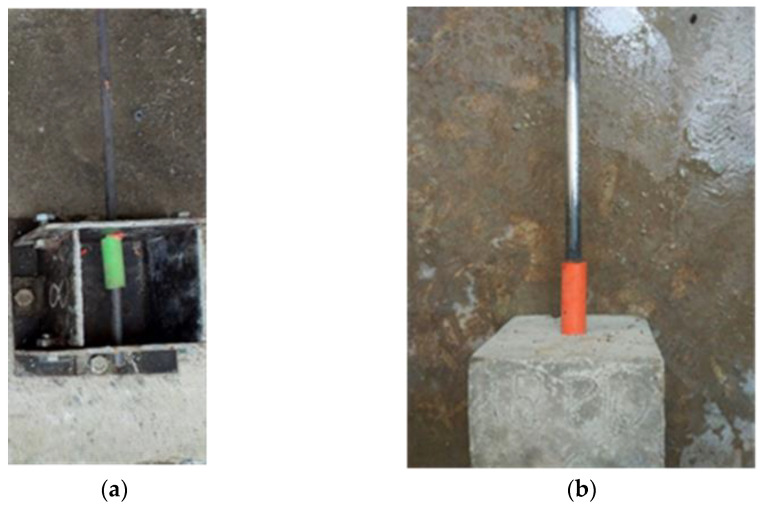

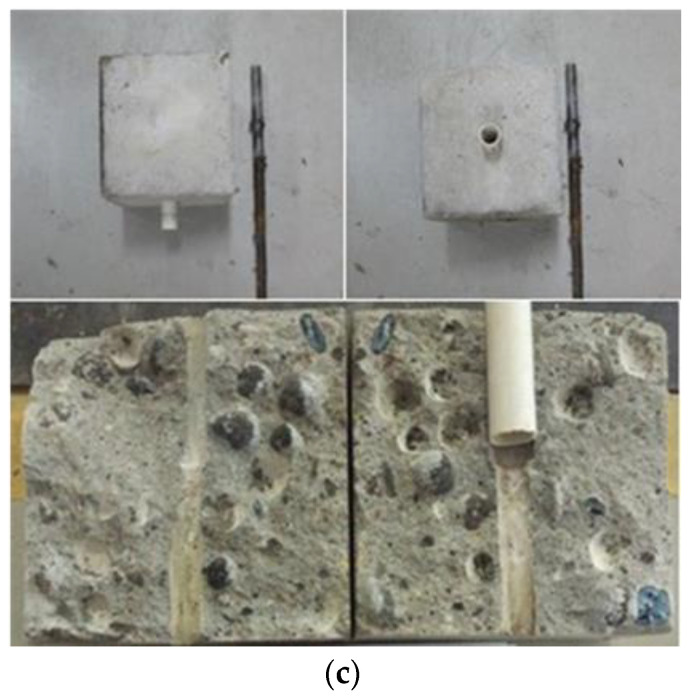
(**a**) Mold and steel rebar. (**b**) Specimen example and (**c**) general pull-out failure pattern in PWCA concrete.

**Figure 9:**
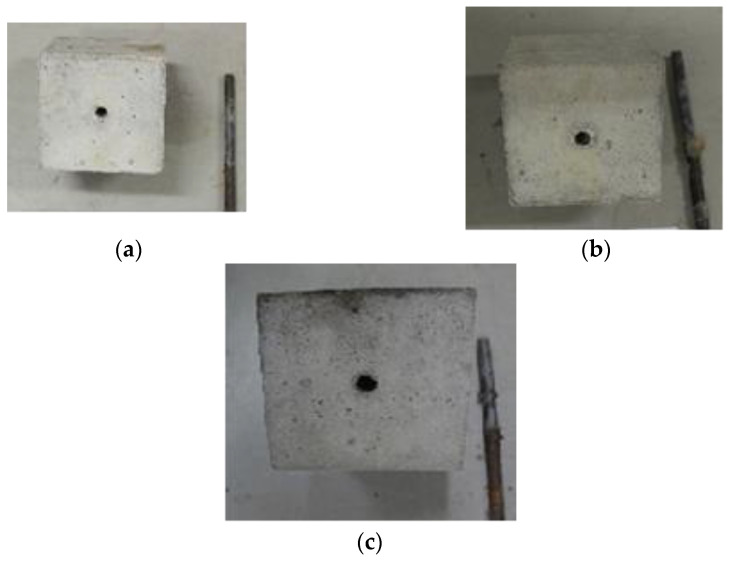
Pull-out failure pattern in PWCA concrete: (**a**) rebar 10 mm, (**b**) rebar 12 mm, and (**c**) rebar 16 mm.

In general, there are two patterns of bond failure in the pull-out test, i.e., splitting- failure and pull-out failure. In this study, all the test specimens failed according to the pull-out failure mode. The mold, specimen example, and general pull-out failure pattern are shown in Figure 8. After the test, the plain bar was separated from the concrete cube and then the cube was split in two parts to better see what happened inside the cube. This is shown in Figure 8c. When the applied load increased gradually, the slip showed a rapid growth as the load approached the maximum, and then the plain rebar was pulled out. At the same time, the slip increased very rapidly, and thereafter the test specimen failed in the form of the detachment of the rebar from the concrete. For the test specimen, the area around the plain rebar did not show any cracks on the concrete surface due to the applied load increasing gradually until it reached the maximum load. Examples of the failure pattern for the 10 mm, 12 mm, and 16 mm rebar are shown in Figure 9. The failure pattern that occurs in plain rebar embedded in plastic waste aggregate concrete coated with sand shows the same results as the failure pattern that occurs in plain rebar embedded in normal concrete as carried out by Xing et al. [37], where the failure that occurs is a pull-out failure.

### 3.4. Bond–Slip Model

Past researchers have developed many bond–slip models for bars embedded in concrete [12,37,39]. The model consists of three segments, namely the ascending portion, linearly descending portion, and residual stress portion.

#### 3.4.1. Proposed Bond Strength

In this study, the bond stress distribution along the rebar embedded in the concrete was assumed to be uniform so that the average bond strength could be determined by an equation [1]. The bond strength for each type of PWCA concrete specimen was obtained by averaging the bond strength of three identical samples to get reliable results. Meanwhile, PWCA concrete specimens that failed by yielding of the rebar were excluded when calculating the bond strength.

**Figure 10:**
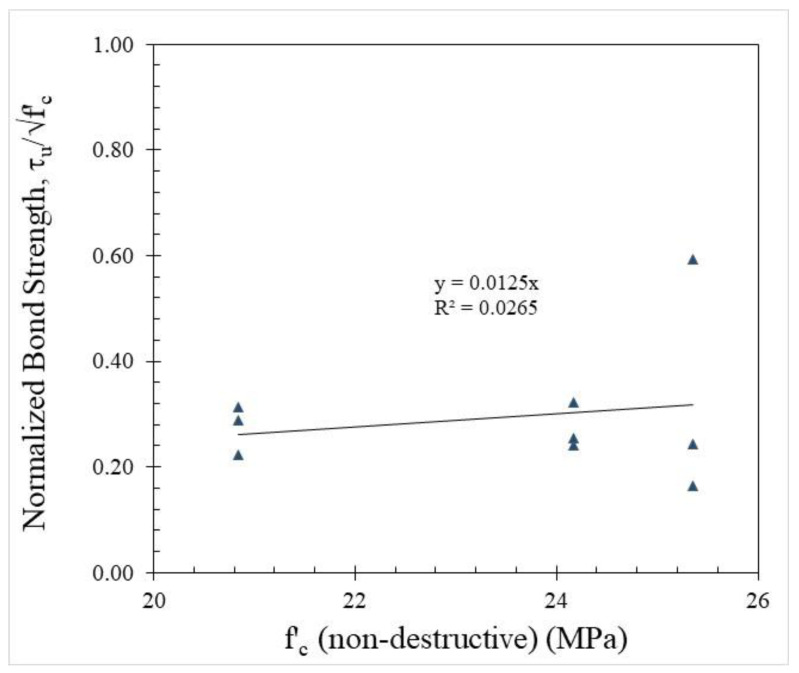
Normalized bond strength vs. compressive strength from UPV test.

Parameters including ultimate bond strength and slip at the time of ultimate bond strength and residual bond strength obtained from the pull-out test measurements were used to characterize the bond–slip relationship. Meanwhile, to eliminate the effect of compressive strength on the bond strength of PWCA concrete, normalization of the bond stress τ_u_/√f_c_ was carried out on all test specimens as per Feldman and Bartlett [8] and Verderame et al. [40]. The normalized bond strength obtained ranged from 0.20 to 0.70, which is lower than the 2.5 value recommended by MC2010 [41] for normal concrete, as shown in Figure 10. Using linear regression between the bond and concrete compressive strength, the obtained coefficient of R^2^ was low. As the value of the Pearson correlation was 0.34 and a weak evidence condition was given the by *p*-value of 0.095, the increase in the bond strength caused by an increase in the concrete compressive strength was not statistically significant. According to Xing et al. [37] and Liu et al. [13], the increased compressive strength due to an improvement in the transition zone between the aggregate and cement matrix and the confinement effect can increase the normalized bond strength of lightweight aggregate concrete.

**Figure 11:**
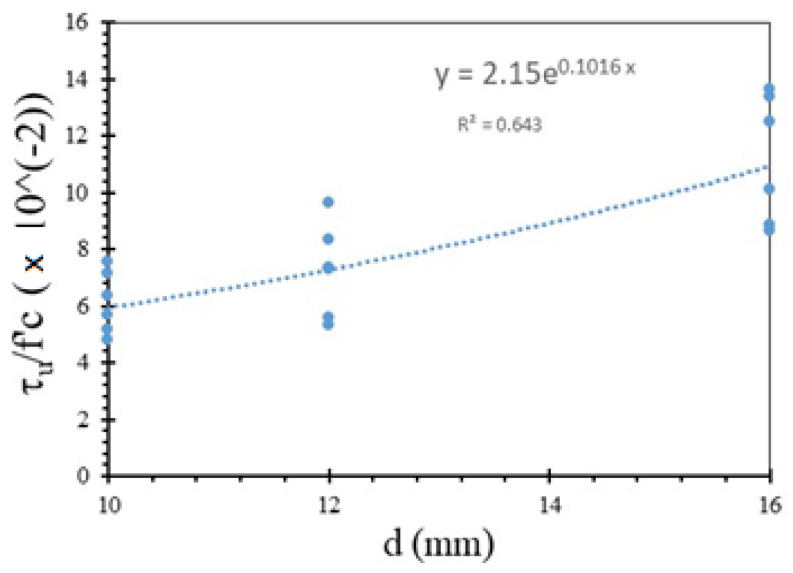
Variation with respect to τ_u_/f′_c_ and diameter.

The rebar diameter also influences the bond strength. Based on the presented data in Figure 11, the bond strength tended to increase with the increasing bar diameter, especially between the 10 mm versus 16 mm rebar diameter embedded in the LC20, LC23, and LC26 PWCAC. The 12 mm rebar diameter gave a lower bond strength for the LC20 and LC23 PWCAC, probably due to the placement of the LVDT sensor, which was not rigidly supported as this concrete group was the first tested. This result is different from previous studies, where a smaller bar diameter can provide a higher strength [42,43,44,45]. However, in this study, the 12 mm and 16 mm diameter of the plain rebar embedded in the LC26 concrete showed a better bond strength than the 10 mm bar diameter. These results are in line with those of Liu et al. [13], who stated that the bond strength increases with the increasing diameter of the reinforcement embedded in lightweight concrete.

**Figure 12:**
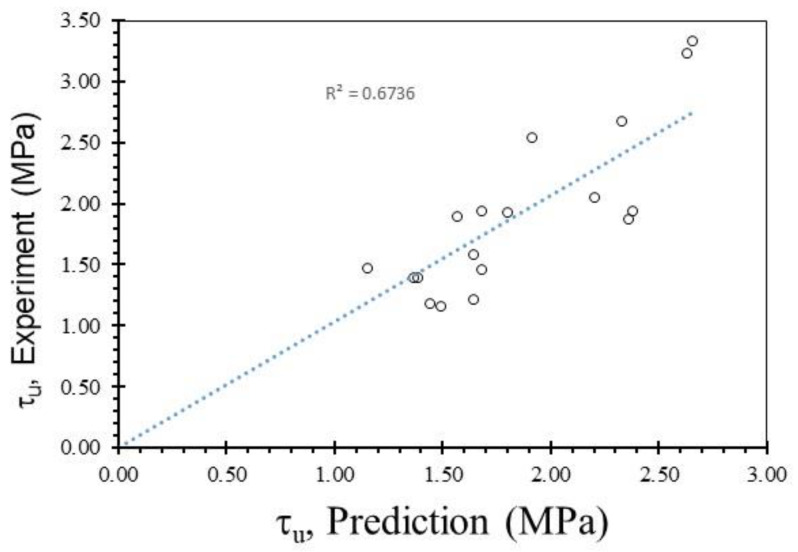
Comparison between the bond strength estimated by the proposed equation and test results.

The bond strength of the rebar embedded in PWCA concrete was determined by analyzing the pull-out test results on specimens with different concrete strengths and reinforcement diameters. Exponential regression analysis was performed on eighteen pull-out test samples to predict the bond strength of plain rebar embedded in PWCA concrete. In the regression analysis, “d” was chosen as the independent variable, as shown in Figure 11, because it is the one that has the most influence on the bond strength of the concrete test specimen, among other parameters that influence it. As shown in Figure 11, the coefficient of R^2^ obtained was 0.643. The regression analysis, as shown in Figure 11, that predicted the bond strength is presented in Equation (2) as follows:

(2) $$\tau_{u}=\left(2.15e^{0.1016\;d}\right)f_{c}^{\prime }$$

where *τ_u_* is the ultimate bond strength, *d* is the rebar diameter, and *f_c_′* is the non-destructive compressive strength obtained from the use of ultrasonic pulse velocity on the PWCAC specimens. The comparison between the bond strength estimated by Equation (2) and the test results is shown in Figure 12, where R^2^ is equal to 0.6736.

#### 3.4.2. Bond–Slip Relationship

Figure 3, Figure 4 and Figure 5 show two graphs of the bond stress–slip relationship. The first graph consists of ascending and descending curves. Besides those two curves, the second graph has a small horizontal line that connects the peaks of the ascending and the beginning of the descending curves. Based on these two patterns, two existing equations were applied in this study that express the relationship between the bond–slip for plain rebar embedded in the sand-coated PWCAC.

**Figure 13:**
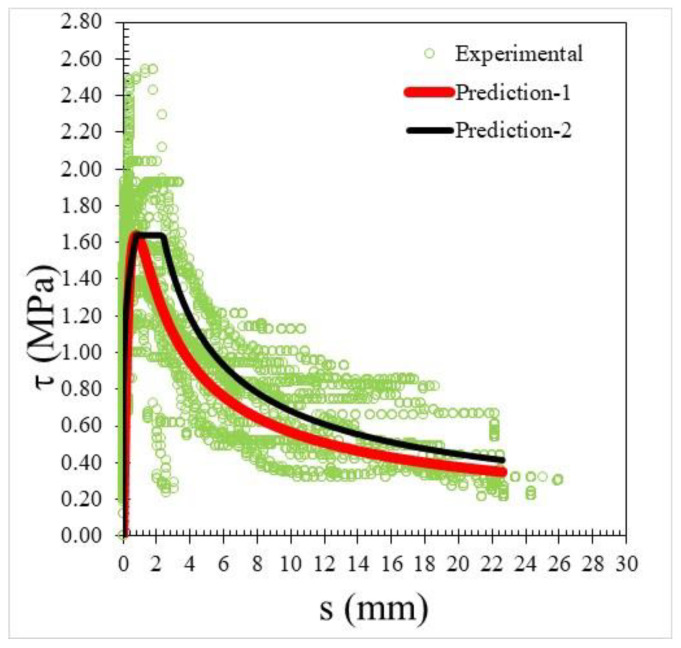
Bond–slip relationship using two prediction formulas vs. experimental results.

The first pattern form of the bond–slip relationship curve was developed based on the stress–strain relationship proposed by Popovics [46], which is shown in Equation (3). The bond–slip relationship equation in MPa is

(3) $$\tau =\tau_{u}\;\left[\frac{s}{s1}\cdot \frac{1.6}{\left(1.6-1\right){\left(\frac{s}{s1}\right)}^{1.6}}\right]$$

where *τ* is the bond stress, *τ_u_* is the ultimate bond strength, *s* is the slip measured at the loaded end during the test (mm), and *s*1 is the slip corresponding to the ultimate bond strength. The prediction results of the bond–slip relationship are shown in the red line curve in Figure 13 where more than 30% of the test results are plotted in the figure, but only *τ_u_*, equal to 1.60 MPa, was directly used for the comparison with another equation, as presented in the following paragraph, without the use of Equation (2).

Meanwhile, the form of the second pattern curve is based on the equation which is identical to the initial part for ribbed bars given in MC2010 [41], as shown by the black line curve in Figure 13. The modified formula given by Equation (6) from Rafi (2019) [39] is similar to Equations (4) and (5) presented below. At the initial stage, according to Equations (4) and (5), the occurring displacement is less than the limit value of *S*_1_.

(4) $$\tau \left(s\right)=\tau_{\mathrm{maks}}{\left(\frac{S}{S_{1}}\right)}^{\alpha }$$

For 0 ≤ *S* ≤ *S*_1_, based on the relationship that *τ_b_* ≈ 0.6 *τ_u_* when *S* = *S*_1_/10, α = 0.2 gives a reasonable representation of the ascending branch. Furthermore, until the displacement reaches the second limit value of *S*_2_, the function is constant at the value interpreted as the ultimate bond strength *τ_u_*.

(5) $$\tau \left(s\right)=\tau_{\mathrm{maks}}{\left(\frac{S}{S_{1}}\right)}^{\alpha_{f}}$$

For slip *S* > *S*_2_, the value of α*_f_* = −0.6 is used, which gives a representation of the descending branch and a residual bond stress of 63% of the peak value when *S* = 10*S*_1_.

**Figure 14:**
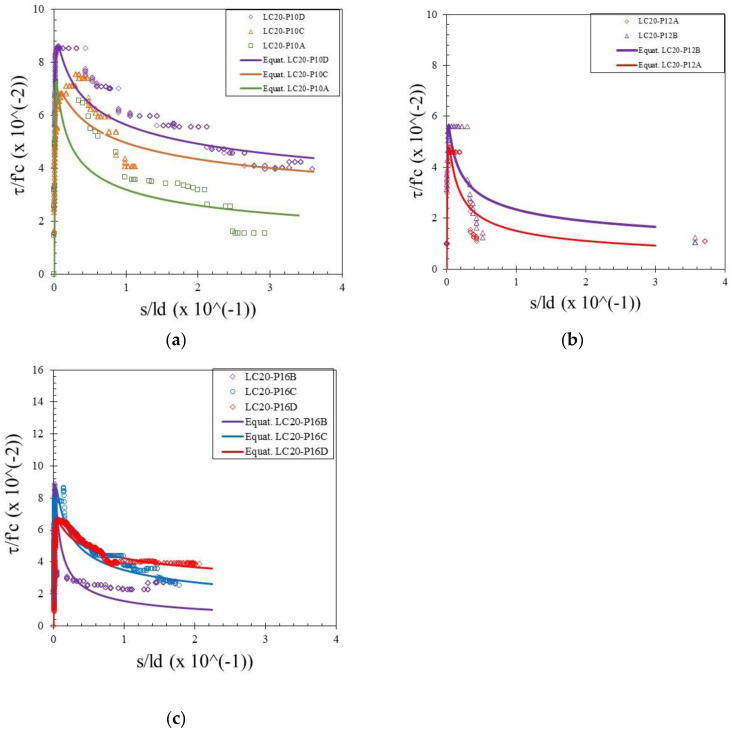
Local bond stress–slip curve for LC20 PWCAC, (**a**) 10 mm plain rebar, (**b**) 12 mm plain rebar, (**c**) 16 mm plain rebar.

**Figure 15:**
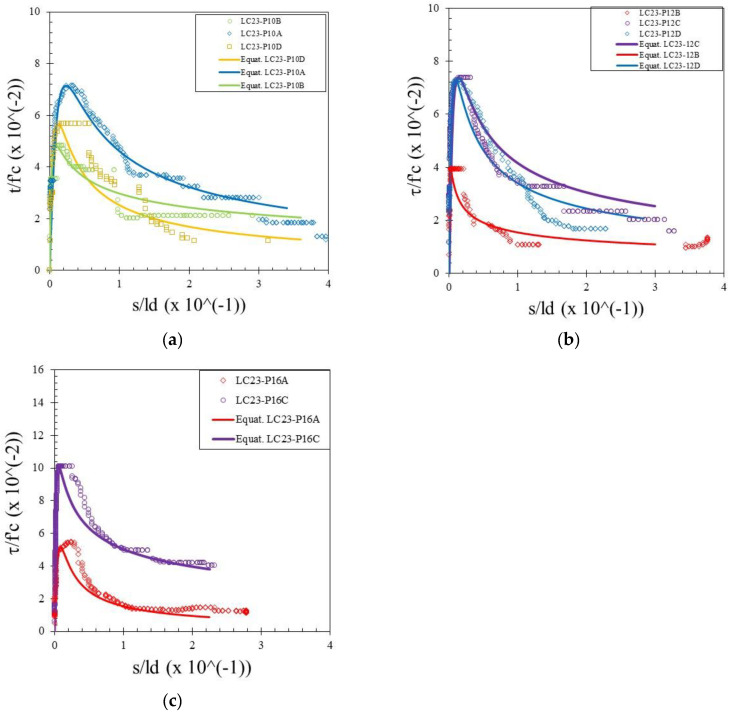
Local bond stress–slip curve for LC23 PWCAC, (**a**) 10 mm plain rebar, (**b**) 12 mm plain rebar, (**c**) 16 mm plain rebar.

**Figure 16:**
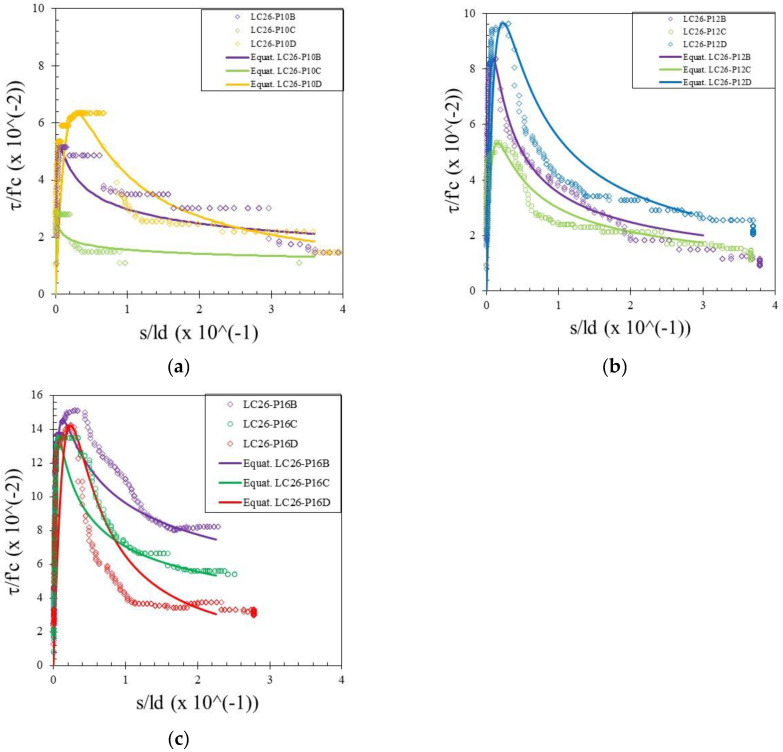
Local bond stress–slip curve for LC26 PWCAC, (**a**) 10 mm plain rebar, (**b**) 12 mm plain rebar, (**c**) 16 mm plain rebar.

By comparing the curves provided by the two-prediction formulas and the experimental results, Equation (3) gives better descending curves then those of Equations (4) and (5). In this regard, bond–slip curves predicted by Equation (3) were plotted against the LC20, LC23, and LC26 PWCAC test results. As previously presented in Figure 4, Figure 5, Figure 6 and Figure 7, the bond stress and slip in Figure 14, Figure 15 and Figure 16 are in the dimensionless form. The predicted curves by using Equation (3), which considers Equation (2), are, in general, in close agreement with the test results.

## 4. Conclusions

An experimental study covering a pull-out test of plain rebar embedded in concrete with sand-coated polypropylene waste coarse aggregate was presented. The concrete quality is represented by three compressive strengths and three rebar diameters that are usually used in the construction industries. The proposed ultimate bond strength of the concrete is derived using an exponential regression analysis. In conjunction with the proposed ultimate bond strength, two existing models derived previously by Popovics [46] and MC2010 [41] could be used to predict the bond–slip curves for plain rebar embedded in this lightweight concrete. However, the Popovics [46] model gave better overall ascending and descending curves compared to the experimental results.

## Figures and Tables

**Table 1:** Physical properties of fine and coarse aggregates.

	RS	PWCA
Density (g/cm^3^)		0.535
Specific gravity:		
Saturated surface dry (SSD)	2.70	1.092
Bulk	2.59	1.071
Apparent	2.90	1.095
Absorption (%)	4.17	2.040
Abrasion (%)	-	10.80
Fineness modulus	2.32	4.25

**Table 2:** Properties of the plain rebar.

Diameter of Rebar, d (mm)	Yield Stress, F_y_ (MPa)	Ultimate Stress, F_u_ (MPa)
10	313	456
12	332	474
16	315	462

**Table 3:** Properties of the mix proportions of PWCAC and normal concrete (NC).

Series	W/C	Cement	Sand	PWCA	Water	Superplasticizer (%)
LC-20	0.29	1	2	2.6	0.91	0.6
LC-23	0.29	1	2	2.0	0.90	0.7
LC-26	0.29	1	2	1.8	0.90	0.8
NC-23	0.30	1	1.3	2.3	0.90	0.7

**Table 4:** Concrete physical and mechanical properties obtained from destructive test.

Series	Sample Number	Density (gr/cm^3^)	Concrete Strength (MPa)
LC-20	I	1.734	20.72
	II	1.838	23.93
	III	1.692	19.88
	IV	1.751	19.49
	Average	1.754	21.01
LC-23	I	1.728	24.13
	II	1.820	26.52
	III	1.784	22.40
	IV	1.811	20.75
	Average	1.786	23.45
LC-26	I	1.738	26.55
	II	1.819	26.32
	III	1.777	26.98
	IV	1.839	26.49
	Average	1.793	26.58

**Table 5:** PWCAC physical and mechanical properties obtained from non-destructive test, and bond strength–slip data from pull-out test.

Series	Bar Dia.	Density (gr/cm^3^)	Concrete Strength (MPa)		Bond Strength	Slip
			Non-Destructive	Average	Bond Strength (MPa), τ_u_	Free end Slip (mm), S_1_
LC26-P10B	10	1.889	28.33	25.36	1.46	0.28
LC26-P10C	10	1.913	27.69		0.80	0.02
LC26-P10D	10	1.871	30.35		1.93	1.43
LC26-P12B	12	1.779	23.12		1.93	0.54
LC26-P12C	12	1.819	22.63		1.20	0.90
LC26-P12D	12	1.787	26.35		2.54	1.36
LC26-P16B	16	1.782	24.36		3.68	2.39
LC26-P16C	16	1.807	24.13		3.25	0.55
LC26-P16D	16	1.773	21.31		3.03	1.90
LC23-P10A	10	1.867	26.42	24.17	1.89	1.15
LC23-P10B	10	1.826	24.33		1.17	0.55
LC23-P10D	10	1.780	27.69		1.57	0.61
LC23-P12B	12	1.807	31.31		1.23	0.13
LC23-P12C	12	1.695	18.85		1.39	0.81
LC23-P12D	12	1.754	19.03		1.39	0.57
LC23-P16A	16	1.877	25.50		1.40	1.90
LC23-P16C	16	1.703	20.20		2.04	0.47
LC20-P10A	10	1.706	22.23	20.84	1.62	0.137
LC20-P10C	10	1.687	19.42		1.47	1.52
LC20-P10D	10	1.622	20.31		1.75	0.25
LC20-P12A	12	1.723	21.67		1.04	0.19
LC20-P12B	12	1.750	20.58		1.15	0.16
LC20-P16B	16	1.796	21.81		1.93	0.14
LC20-P16C	16	1.775	21.60		1.87	1.17
LC20-P10A	10	1.706	22.23		1.62	0.137

**Table 6:** Normal concrete physical and mechanical properties obtained from non-destructive test and bond strength–slip data from pull-out test.

Series	Bar Dia.	Density (gr/cm^3^)	Concrete Strength (MPa)		Bond Strength	Slip
			Non-Destructive	Average	Bond Strength (MPa), τ_u_	Free and Slip (mm), S_1_
NC23-P16A	16	2.330	22.33	22.96	2.39	0.85
NC23-P16B	16	2.363	23.35		1.53	2.11
NC23-P16C	16	2.388	23.21		2.13	0.36

## Data Availability

The data presented in this study are available on request from the corresponding author.

## References

[1] Cairns J. (2020). Local bond–slip model for plain surface reinforcement. Struct. Concr..

[2] Tang C.-W., Cheng C.-K. (2020). Modelling Local Bond Stress–Slip Relationships of Reinforcing Bars Embedded in Concrete with Different Strengths. Materials.

[3] (2003). Bond and Development of Straight Reinforcing Bars in Tension.

[4] Mo Y.L., Chan J. (1996). Bond and Slip of Plain Rebars in Concrete. J. Mater. Civ. Eng..

[5] Feldman L., Bartlett F.M. (2005). Bond Strength Variability in Pullout Specimens with Plain Reinforcement. ACI Struct. J..

[6] Zhang X., Wu Z., Zheng J., Dong W., Bouchair A. (2016). Ultimate bond strength of plain round bars embedded in concrete subjected to uniform lateral tension. Constr. Build. Mater..

[7] Ahmad S., Pilakoutas K., Rafi M.M., Khan Q.U.Z., Neocleous K. (2018). Experimental Investigation of Bond Characteristics of Deformed and Plain Bars in Low Strength Concrete. Sci. Iran..

[8] Feldman L., Bartlett F.M. (2007). Bond Stresses along Plain Steel Reinforcing Bars in Pullout Specimens. ACI Struct. J..

[9] Alkaysi M., El-Tawil S. Bond between Ultra-High Performance Concrete and Steel Bars. Proceedings of the First International Interactive Symposium on UHPC.

[10] Shang H.-S., Cui F.-K., Zhang P., Zhao T.-J., Ren G.-S. (2017). Bond behavior of steel bar embedded in recycled coarse aggregate concrete under lateral compression load. Constr. Build. Mater..

[11] Xiao J., Falkner H. (2007). Bond behaviour between recycled aggregate concrete and steel rebars. Constr. Build. Mater..

[12] Hossain K.M.A. (2008). Bond characteristics of plain and deformed bars in lightweight pumice concrete. Constr. Build. Mater..

[13] Liu X., Liu Y., Wu T., Wei H. (2020). Bond-slip properties between lightweight aggregate concrete and rebar. Constr. Build. Mater..

[14] Teo D.C.L., Mannan M.A., Kurian V.J. (2007). Structural Bond Performance of Lightweight Concrete. Build. Environ..

[15] Purnomo H., Pamudji G., Satim M. (2017). Influence of uncoated and coated plastic waste coarse aggregates to concrete compressive strength. MATEC Web Conf..

[16] Pamudji G., Satim M., Chalid M., Purnomo H. (2020). The Influence of River and Volcanic Sand as Coatings on Polypropylene Waste Coarse Aggregate towards Concrete Compressive Strength. J. Teknol..

[17] Abu-Saleem M., Zhuge Y., Hassanli R., Ellis M., Rahman M., Levett P. (2021). Stress-Strain Behaviour and Mechanical Strengths of Concrete Incorporating Mixed Recycled Plastics. J. Compos. Sci..

[18] Al Bakri A.M.M., Tamizi S.M., Rafiza A.R., Zarina Y. (2011). Investigation of HDPE plastic waste aggregate on the properties of concrete. J. Asian Sci. Res..

[19] Abeysinghe S., Gunasekara C., Bandara C., Nguyen K., Dissanayake R., Mendis P. (2021). Engineering Performance of Concrete Incorporated with Recycled High-Density Polyethylene (HDPE)—A Systematic Review. Polymers.

[20] Choi Y.W., Moon D.J., Kim Y.J., Lachemi M. (2009). Characteristics of mortar and concrete containing fine aggregate manufactured from recycled waste polyethylene terephthalate bottles. Constr. Build. Mater..

[21] Frigione M. (2010). Recycling of PET bottles as fine aggregate in concrete. Waste Manag..

[22] Islam J., Meherier S., Islam A.R. (2016). Effects of waste PET as coarse aggregate on the fresh and harden properties of concrete. Constr. Build. Mater..

[23] Alqahtani F. (2021). Sustainable Green Lightweight Concrete Containing Plastic-Based Green Lightweight Aggregate. Materials.

[24] Lakshmi R., Nagan S. (2010). Studies on Concrete containing E plastic waste. Int. J. Environ. Sci..

[25] Arora A., Dave U.V. (2013). Utilization of e-waste and plastic bottle waste. Int. J. Stud. Res. Technol. Manag..

[26] Ali K., Qureshi M.I., Saleem S., Khan S.U. (2021). Effect of waste electronic plastic and silica fume on mechanical properties and thermal performance of concrete. Constr. Build. Mater..

[27] Ahmad F., Jamal A., Mazher K.M., Umer W., Iqbal M. (2022). Performance Evaluation of Plastic Concrete Modified with E-Waste Plastic as a Partial Replacement of Coarse Aggregate. Materials.

[28] Ullah S., Qureshi M.I., Joyklad P., Suparp S., Hussain Q., Chaiyasarn K., Yooprasertchai E. (2022). Effect of partial replacement of E-waste as a fine aggregate on compressive behavior of concrete specimens having different geometry with and without CFRP confinement. J. Build. Eng..

[29] Ekolu S.O. (2020). Service Life Design for Inland Concrete Structures in Africa: Kampala Case Study. RILEM Bookseries.

[30] Pamudji G., Heribowo B., Adam Y., Purnomo H. (2018). Bond-Slip Behavior of Steel Bar Embedded in Lightweight Concrete Using Sand Coated Polypropylene Coarse Aggregate. Mater. Sci. Forum.

[31] (1994). RC 6 Bond Test for Reinforcement Steel. Pull-Out Test. RILEM Technical Recommendations for the Testing and Use of Construction Materials.

[32] Pamudji G., Purnomo H., Katili I., Imran I. The use of plastics waste as coarse aggregates. In Proceeding of the 6th Civil Engineering Conference in Asia Region: Embracing the Future through Sustainability.

[33] (2000). Standard Specification for Lightweight Aggregates for Structural Concrete.

[34] (2014). Standard Specification for Lightweight Aggregates for Structural Concrete.

[35] (2004). Committee Test Method for Determining Density of Structural Lightweight Concrete.

[36] (2004). Standard Test Method for Compressive Strength of Cylindrical Concrete Specimens.

[37] Xing G., Zhou C., Wu T., Liu B. (2015). Experimental Study on Bond Behavior between Plain Reinforcing Bars and Concrete. Adv. Mater. Sci. Eng..

[38] Wang L., Song Z., Yi J., Li J., Fu F., Qian K. (2019). Experimental Studies on Bond Performance of BFRP Bars Reinforced Coral Aggregate Concrete. Int. J. Concr. Struct. Mater..

[39] Rafi M.M. (2019). Study of Bond Properties of Steel Rebars with Recycled Aggregate Concrete. Analytical Modeling. Strength Mater..

[40] Verderame G.M., DE Carlo G., Ricci P., Fabbrocino G. (2009). Cyclic bond behaviour of plain bars. Part II: Analytical investigation. Constr. Build. Mater..

[41] FIB (2010). FIB Model Code 2010.

[42] Maree A.F., Riad K.H. (2014). Analytical and experimental investigation for bond behaviour of newly developed polystyrene foam particles’ lightweight concrete. Eng. Struct..

[43] Kim D.-J., Kim M.S., Yun G.Y., Lee Y.H. (2013). Bond strength of steel deformed rebars embedded in artificial lightweight aggregate concrete. J. Adhes. Sci. Technol..

[44] Ichinose T., Kanayama Y., Inoue Y., Bolander J. (2004). Size effect on bond strength of deformed bars. Constr. Build. Mater..

[45] Gambarova P.G., Rosati G.P., Zasso B. (1989). Steel-to-concrete bond after concrete splitting: Constitutive laws and interface deterioration. Mater. Struct..

[46] Popovics S. (1973). A numerical approach to the complete stress-strain curve of concrete. Cem. Concr. Res..

